# Improving Health Outcomes of Children through Effective Parenting: Model and Methods 

**DOI:** 10.3390/ijerph110100296

**Published:** 2013-12-23

**Authors:** Martha Okafor, Daniel F. Sarpong, Aneeqah Ferguson, David Satcher

**Affiliations:** 1 Morehouse School of Medicine, Satcher Health Leadership Institute, 720 Westview Drive, Southwest Atlanta, GA 30310, USA; E-Mails: aferguson@msm.edu (A.F.); dsatcher@msm.edu (D.S.); 2 RTRN-Data Coordinating Center, Jackson State University, 1230 Raymond Road, Jackson, MS 39204, USA; E-Mail: dsarpong@jsums.edu

**Keywords:** parenting, smart and secure children, child development, emotional wellbeing, mental health, community-based participatory research, conversepedia

## Abstract

This article reports on the design, development, testing and presentation of preliminary evidence of a translational, culturally relevant parenting education model, titled Smart and Secured Children (SSC). SSC, a quality parenting curriculum, prepares disparate African American parents as leaders for transforming their parenting behaviors and leading their peers and community in changing existing parenting culture. The article recommends expanded utility of identified promising processes, approaches and practices to engage African American parents to lead in addressing health inequity conditions in their families and communities. It adds to the growing scientific literature on the association between parent–child relationship quality and a wide variety of children physical, emotional and social outcomes. SSC applied principles of developmental theories; community based participatory research (CBPR), and iterative Delphi method between the community stakeholders, parents and researchers. The delivery approach of SSC was revamped from professional-led to parent-led content presentation and delivery methods using a conversational learning approach, referred to as ‘*conversepedia*’. Parents’ leadership development training and delivery of this curriculum in social supportive groups improved their mental wellbeing, parenting capacity and leadership skills. Parents do matter and can choose positive influence in their lives and are capable of reversing negative peer influence.

## 1. Introduction

Low socioeconomic status (SES) and chronic poverty negatively impact the health and well-being of individuals and families [1]. Low SES is positively, particularly in ethnic racial minority populations, associated with an increased risk of morbidity and mortality and prevalence of diagnosable mental disorders [2,3,4,5,6,7]. Research also demonstrates a strong association between chronic poverty and disparities in child health mental status, educational attainment and general mental health, behavioral health and intellectual growth and development [8,9,10]. “Inequities in health arise because of inequities in society—in the conditions in which people are born, grow, live, work, and age” [11].

Evidence from neuroscience research suggests that exposure to cumulative negative environmental, social and behavioral risk factors creates adverse childhood experiences that are toxic to the developing brain of a child and may lead to underdeveloped neural connections or weakened brain architecture which are necessary for building the competencies for successful navigation of adulthood [12,13,14]. There is indication that quality parenting, during early childhood, can mitigate up to 50% of the negative impact of poverty on a child’s mental and behavioral development [15,16]. Several studies have demonstrated significant associations between the quality of the parent-child relationship and wide variety of physical, emotional and social outcomes in children [17,18,19,20,21,22]. However, while there are studies that have examined parenting strengths among African Americans (AAs), most of the existing parenting education curricula being used by AA families are not developed with active participation of AA mothers, fathers, siblings, caregivers and/or grandparents [23,24,25,26,27]. 

In response to this significant gap in knowledge in the literature, the Satcher Health Leadership Institute at Morehouse School of Medicine partnered with several local communities, to develop and implement the Smart and Secured Children (SSC). A peer facilitated, parenting curriculum, designed to coach parents to be empowered to build the capacity within themselves and their communities to reduce early childhood disparities in mental health and educational attainment [28].

This article first describes the process of developing a “parent-centered”, community-driven parenting curriculum, known as SSC parenting curriculum, aimed at increasing knowledge about child development, particularly brain development, and empower mothers and fathers of all ages to become parents who are nurturing smart and secured children. The pilot-testing phase of the SSC parenting curriculum afforded the SSC investigators and personnel to learn of the strengths and challenges of parenting among vulnerable African Americans, as well as plan, develop and test the content validity of the SSC curriculum with the parents whose parenting behaviors were hypothesized to change through the SSC intervention. Data on the efficacy of the intervention is preliminary and brief. More extensive data driven article will be disseminated at a later date. 

## 2. Design and Methods

### 2.1. Design and Methods for Developing the Smart and Secured Children Parenting Curriculum

Several key principles of community-based participatory research (CBPR) served to guide this academic-community partnership. High priority was given to: implementing SSC curriculum development as a cyclical process, using an iterative Delphi method and collaborative partnership between community stakeholders and parents, and academic partners at all phases of curriculum development; building on the existing strengths and resources of the community; promoting co-learning; integrating ecological perspective in improving child health and wellbeing; and sharing the knowledge gleaned along the way with all stakeholders. 

Parents (defined in this study as the consistent adult who is caring for the child, including birth, foster and adopted parents; the kith and kin and grand-parents) who live, work, play and active in the neighborhoods were recruited into this study. Study participants were recruited through collaborative efforts with the neighborhoods’ early care learning, childcare, food pantries, libraries, and not-for-profit, governmental and faith-based organizations that distributed our fliers and referred parents to our study. Parents were engaged in this study as we conducted: (a) three Meet and Greet sessions in the three targeted zip codes with 22 participants; (b) nine parents’ focus group with 88 participants; and (c) Village Meeting with more than 75 participants (individuals may have participated in more than one activity, for example, a village meeting and a focus group). Forty (40) parents worked with us to develop the Smart and Secure Children (SSC), test its relevance and application in their daily routines, as well as validate the effectiveness of its delivery approach using peers as mentors and leaders.

Attachment Theory [29], Social Learning Theory [30] and Attribution Theory [31] provide the theoretical framework for the construction of the SSC parenting curriculum. The quality attachment (initial parent-infant relationship and on-going parent-child relationship) articulated in the Attachment Theory, is embedded in SSC curriculum. By developing a curriculum with a foundation in Attachment Theory, SSC parent participants learned how adults can form quality connections and relationships with infants and children and raise emotionally healthy children. The Social Learning Theory reflected in SSC curriculum content informs SSC social participation learning model. Lastly, the Attribution Theory is centered on how people understand the causes of their behaviors and other people’s behaviors. 

### 2.2. Design and Methods for Pilot Testing of the Smart and Secured Children Parenting Curriculum

*Study Design:* We used a pre-test and post-test study design to assess changes and outcomes among parent participants from baseline to post-intervention. The intervention included a 10-module training parenting curriculum, which uses a three-tiered, train-the-trainer model where level 1 is the parent mentors (PM); level 2, the parent leaders (PL); and level 3, the parent peer learners (PPL). Studies have revealed that the use of ‘peers’ in behavior modification and life-style change is very effective and that “leadership” is one of the determining factor of success across families, neighborhoods, societies and organizations [14,15]. At level 1, the parent mentor was recruited to mentor and guide the development of the parent leader. They also co-train the parent leaders on the SSC curriculum and observe and support the parent leaders as they facilitate conversational learning in the SSC sessions. Once trained, the parent leader then becomes the “trainer” and goes on to facilitate the 10-module SSC curriculum among a group of their parent peers. In addition to facilitating parenting sessions, parent leader coach and help the parent peer-learners to make incremental changes and improvements in their parenting beliefs, attitude and behaviors. Level 3 are the “parent peer learners”, who come together to share and learn practical things they can do at home every day with their children and families to nurture and raise smart and secure children ready for school at age five. 

*Target Population:* The SSC project targeted low-income African American families with children ages five and under, from Atlanta neighborhoods who are faced with negative social determinants, such as poverty, incarceration, involvement in child welfare, homeless, and at high risk of serious mental distress or mental illness according to Annie Casey Report. These conditions can have significant negative impact on parenting ability and create adverse childhood experience. However, for this paper, the target population was the parents since the pilot testing phase of the intervention was designed to evaluate the efficacy of changes in the parents’ knowledge of child development, life style behaviors and leadership. 

*Recruitment:* Recruitment for the Pilot Study was conducted using snowball sampling and social network methods. The first participants of SSC were the parent mentors (PMs)—who performed the role of mentoring the parent leaders. The second wave of parent participants was the parent leaders (PL), who in turn recruited the next wave, the peer parent learners (PPL). All parents’ participants (PL and PPL) had the same underlying inclusion criteria. Some parents were recruited to participate as parent leaders (PLs)—which means they were to receive leadership training in addition to SSC training so that they can go on to train other parents. The third and final group recruited was the peer parent learners (PPLs), and they received SSC intervention. Recruitment techniques included but were not limited to outreach at: churches, homeless shelters, food banks, childcare centers, Head Start/Early Head Start, parks and recreation centers, community based human and social services entities, family courts, governmental agencies administering eligible services, such as food stamps, WIC, Medicaid, and pre-school programs, *etc.* Morehouse School of Medicine (MSM) leveraged existing partnerships in the targeted communities, such as the Neighborhood Healthy Child Development Project (NHCDP), Prevention Research Center (PRC) and Atlanta Promise Neighborhood. SSC research team employed the following marketing activities: personal referrals, brochures, flyers, and community presentations. Eligibility criteria for parent leaders (PLs) were: parent participants referred for leadership development; expressed an interest in SSC training; good communication skills; level of interpersonal skills and sensitivity; and had some experience at leading small groups.

*Selection Criteria:* Inclusion criteria for parent participants (PLs or PPLs) are: residence in one of the target communities, work and spend majority of day time in targeted communities, have a child in their direct care aged 0–5, and have a household income at or below 235% federal poverty line (FPL) based on self report and or existing eligibility for public assistance with the same income eligibility criteria. 

*Overview of Intervention*: The SSC curriculum of quality parenting was designed to mitigate negative impact of poverty and build protective and resilient factors for children. The short term goal is to increase parents’ knowledge of child development particularly the social-emotional and neurobiology of the developmental process and how parent-child relationship, nutrition and physical activity impact a child development. The content of the SSC curriculum is described in the next section. 

*Content of SSC Curriculum*: The proposed SSC intervention was to produce communal processes of changing parenting behaviors among vulnerable African American parents with the long-term goal of improving children’s brain development, cognitive functioning, language and reading development, and social and emotional health. The main topics of the ten modules of the SSC curriculum are:


How The Brain Develops
Session 1
What Makes A Child’s Brain Develop and Grow?
Session 2
Watch Them Grow: Developmental Milestones
Session 3
Ways to Help Your Child’s Brain Thrive & Come Alive
Session 4
Brain Thrive & Come Alive: Watching TV Together!
Session 5
Brain Development Review & Defining Social and Emotional Health
Session 6
Critical Needs of Socially and Emotionally Healthy Children
Session 7
Ten Things to Do at Home Towards Social and Emotional Health
Session 8
Parents’ Self-Care and Nurturing
Session 9
Working with Challenging Behaviors Through Positive Discipline
Session 10

**Figure 1:**
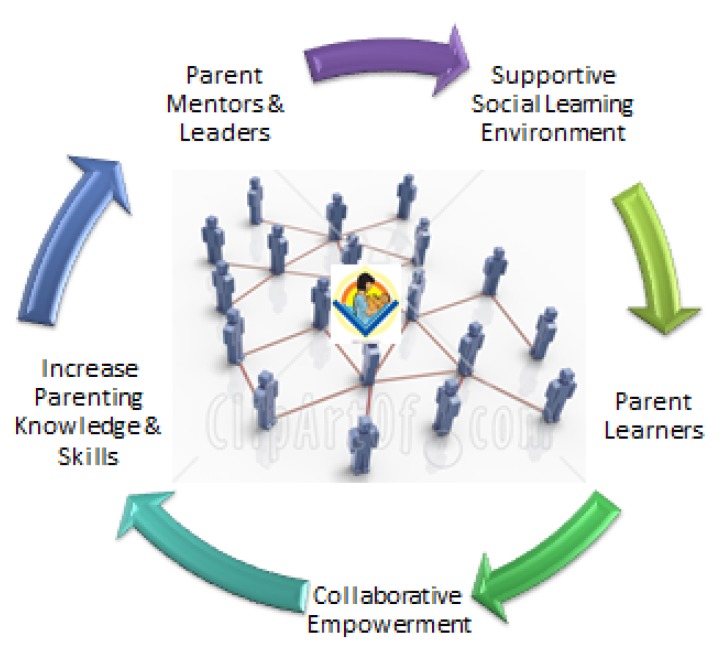
Improving parenting through conversepedia.

This curriculum builds on culturally centered traditional values of communalism, spirituality and innate desire for leadership among this targeted population of African American parents, and was designed to produce effective methods for conducting parent leadership research for other minority populations that share similar values. The SSC curriculum is only delivered to participating parents using a “conversepedia” approach. “Conversepedia” is derived from two words: “conversation” and “pedagogy” [28,32]. This method uses conversation, which is a natural process people use to share information and learn new knowledge to facilitate learning of quality parenting in groups. Figure 1, depicts the connectivity between parents using collaborative mode of communication “conversation” to promote the five qualities in outer circle. 

*Data Collection and Measurement*: To assess positive mental health, the 14 items of the Mental Health Continuum Short Form [MHC-SF] was used. The MHC-SF is a self-report survey that measures positive mental health by using three subscales: emotional, psychological, and social well-being and has demonstrated good internal reliability in each [33,34]. A battery of data collection tools have been developed by the Satcher Health Leadership Institute’s and the Research Core of the Morehouse School of Medicine’s Center for Applied and Clinical Research, like the SSC Knowledge Assessment Tool. This knowledge assessment tool asks three questions per module and is completely aligned with the specific content in the 10 modules of the curriculum that is split evenly between a focus on early childhood brain development and social emotional health. This SSC knowledge tool uses a multiple-choice format for questions delivered pre and post to assess increased parent knowledge of the role of nutrition, physical activity, creating a stimulating home environment, reading to and hugging a child, stress-reduction, and positive discipline practices on healthy child development cognitively and socially-emotionally. The next tool created by the research team and tested among the parents during the pilot study was the Readiness to Change Tool, designed to capture readiness to change parenting behaviors that do not promote health or prevent disease. The question options for this tool was asked in three categories (nutrition, physical activity, general well-being) with the following five response options: “pre-contemplation”, “contemplation”, “preparation”, “action”, and “maintenance”. Fidelity of the delivery of curriculum by parent leaders (PLs) to their peers was assessed through observation using a checklist with response options “yes”, “no”, “N/A”. This tool was used by the PMs and a research member who functions as the ‘fidelity officer’ for assessing adherence to: time allotment for session, standard use of course materials, providing an overview of course objectives at start of class, appeared well-prepared, and used a manner that was engaging, encouraging and inclusive of all parents. The actual development of the SSC intervention, which is not the focus of this paper, was done through a series of focus groups and iterative feedback mechanism mainly between the researchers and community of parents. Other stakeholders such as: the early care providers, community organizations providing health and human services to the targeted neighborhoods and pediatric healthcare providers provided constructive feedback to the development of SSC intervention.

*Statistical Analysis*: Both quantitative and qualitative methods were used to analyze the data. Since the focus of this article was on the design, model and methods employed in developing and implementing SSC intervention, only basic data is presented in this article. In-depth presentation of the efficacy of the intervention will be presented in subsequent papers or articles among multiple cohorts of SSC parents. Hence, paired T-Test and Chi-square tests were performed to assess a pre- and post difference for continuous measures and categorical measures, respectively. Preliminary content analysis using NVivo 10 (QSR International (Americas) Inc., MA, USA) was conducted to highlight emerging theme derived from the qualitative data, excerpts of journal presentations and responses to survey questions. Descriptive statistics were performed to describe sample characteristics. All significance testing were performed at the 0.05 level and SAS version 9.2(SAS Institute Inc., Cary, NC, USA) was the statistical software package used for data analysis.

## 3. Results

### 3.1. Derived Conceptual Framework—Smart and Secured Children Model

The delivery approach (content presentation and delivery) of typical parenting curriculum is led by professionals in early childhood using traditional lecture format. Parents who participated in SSC emphasized the need to shift from the professional-led and lecture approach to parent led using a conversational learning approach, “*conversepedia*”. Table 1 provides a summary of how SSC parenting curriculum was delivered using “*conversepedia*.” 

**Table 1:** Comparison of Parenting Curriculum Delivered through Traditional method *vs.* Conversepedia.

Traditional Delivery	Conversepedia
The Professionals are experts and have all answers	Everyone is a learner—focus is to facilitate quality learning environment for all learners
The expert professional teaches the parenting and parents are passive and sometimes are involved in questions and answers	The parents trained as leaders facilitate active conversations among parents to help them learn and take small doable actions of success weekly
The setting is a classroom structure	The setting is a circle of up to 6 peer parents
The classroom setting creates win or lose environment with levels of anxiety and insecurity	The supportive social learning setting creates secure collaborative motivation to learn and discover success for continuous improvement
The expert covers the content and associated tests	The parent leaders facilitate conversations on SSC content with parents sharing real life application, experience and solutions
Learning takes time since everyone is taught the same way with some parents learning and others not learning	Learning is intrinsic with parents improving their knowledge, beliefs and values as they self-develop and apply what they learn daily at home and lead their peers in the community to change parenting culture.

#### Community Building by Parents

To build a sense of community, the first generation of SSC parents (parent mentors and their members) formed five tribes. The “Nubians”, the “Pearls of Knowledge”, and the “Butterfly Effect” were the three tribes headed by women. The “Fathers” and the “Men of Vision and Purpose” [MVP] were the two headed by men. Each tribe leader or parent mentor (PM), helped to recruit the parent leaders (PLs) and train them on the SSC curriculum by modeling the ‘conversepedia’ experience. The PL on the other hand, after matriculating through the program helped to recruit the parent peer learners (PPLs), and they trained them on SSC. This approach of building a community of parents utilized a social network model. The geometric growth of a tribe equated the phenomenon in business in the area of network marketing. In a few generations each tribe will become very large as members of each tribe continues to engage their friends and family members in conversations around quality parenting. Thus, the potential impact of using this approach to improve parenting skills is phenomenal. The multiplicative effect of the SSC model in training parents has both social and political implication in empowering a community to seek the best developmental plan for their children.

### 3.2. Results of Pilot Testing

Concisely, the following information was derived from pilot testing the parenting curriculum to develop smart and children. Details of the pilot testing will be presented in a subsequent article.

*Sample Characteristics*: A total of 125 parents in the targeted communities in the metropolitan Atlanta, Georgia Neighborhood Planning Unit (NPU) V were recruited to pilot test the parenting curriculum designed to develop smart and secure children. Of the 125 recruited, 83 (66%) parents actively enrolled in the program. The sites for the training sessions were predominantly three community facilities in the metropolitan Atlanta area. Sixty-four percent of the participants were women (see Table 2). The distribution of educational attainment, employment status, and family income are also presented in Table 2 below. 

**Table 2:** Socio-demographic Characteristics of Sample.

Characteristics	N = 83
Gender, Female (%)	64.0
*Educational Attainment (%)*	
<High School	4.1
High School/GED	28.6
Some College/Associate Degree	40.8
Bachelor’s Degree	16.3
>Bachelor’s Degree	10.1
*Employment Status (%)*	
Full-time	34.0
Part-time	20.0
Temporary Work/> 1 paid job	4.0
Own Business	16.0
Not Employed	26.0
*Family Income (%)*	
<$250	27.0
$250–$499	43.8
$500–$1,000	10.4
>$1,000	18.8

**Figure 2:**
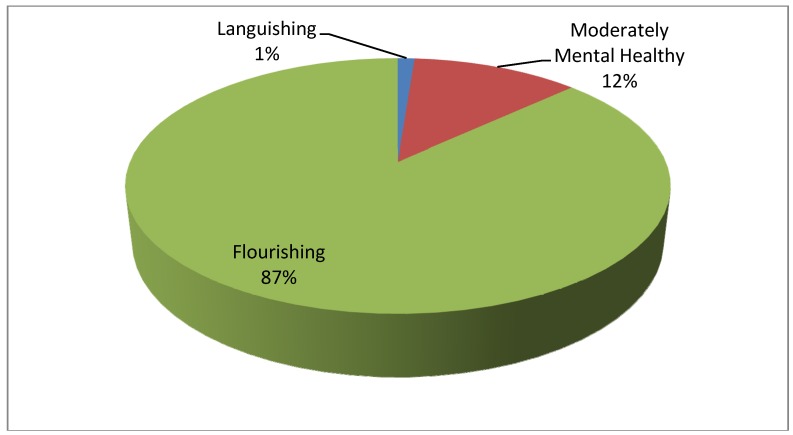
Distribution of the MHC-SF at Baseline (n = 83).

*Mental Health Assessment*: Majority of the participants (87%, Figure 2) in the program were highly motivated and positively strong individuals who are seeking opportunities to improve their parenting skills in raising a smart and secure child. Based on the Quadrant Analysis proposed by Keyes and Lamers *et al.* [33,34] for scoring the Mental Health Continuum Short Form, the data suggest that 87% of the parent participants reported their mental health as flourishing, 12% reported to be moderately mentally healthy, and 1% languishing. Using Keyes’ [33] quadrant analysis, parents who reported moderately mentally healthy condition have low symptoms of a mental illness, while parents who reported languishing mental condition have symptoms of mental illness and poor mental health. 

*Knowledge of Brain and Child Development Assessment:* Though the data are not presented, The comparative analyses indicated that at the pre-test, administrated prior to any instruction, there were gender differences in the scores for: Section 2 (men *vs.* women: 18.1 *vs.* 37.2; p = 0.0299); Section 7 (men *vs.* women: 15.3 *vs.* 47.7; *p* = 0.0013); and Section 10 (men *vs.* women: 5.0 *vs.* 11.9; p = 0.0483). For these three sections, the women significantly scored higher than the men. 

*Leadership Skills Post Intervention*: At the end of the SSC training, all (100%) of the parents surveyed indicated that their leadership skills had improved. Table 3 provides a summary of the main themes that emerged when the parents were asked to list three areas of leadership in parenting that they had improved the most and the three areas that they thought they need to improve the most.

**Table 3:** Summary of Leadership Skills in Parent: After Completing the SSC Training.

Areas of Improvement	Areas of Need Improvement
Communication skills with children	Parent development
Time management	Time management
Developed healthy lifestyle	Getting children to adopt modified behaviors
Creating a safe and conducive learning environment at home	Keeping up with societal changes
Parenting better	Help my children to eat healthy
Share and talk about SSC with family, friends, and people on the bus, train and grocery stores	

### 3.3. Lessons Learned/Recommendations

This pilot project allowed MSM SHLI to test both the curriculum and the processes of implementing the SSC curriculum in the targeted population. Though the project yielded highly positive results, key lessons learned are detailed below:

- Due to very low variability in the 10 post-sessions evaluation, we learned from participants that the burden of data collection was overwhelming and did not add any value to their learning experiences. As a result, 10 post-sessions evaluation will be reduced to three; on the first day; after Part 1 Session 5 (half-way point in training) and the end of Part 2 Session 5.
- Based on the pilot data and input from the parents trained, there was a need to conduct a qualitative study to further the understanding of potential facilitators and barrier to increasing performance on the knowledge acquisition of the training material and self-development given the complexity of the subject matter. As a result of this lesson learned, the SSC parent graduates of the pilot study worked with SHLI to identify areas that needed improvement and delivery changes to optimize learning. These improvements will be made to the SSC content and delivery approach that will be implemented in the full demonstration phase of SSC that is scheduled to follow this pilot test
- Streamline data collection; explore use of technology, such as: mobile or web-technology, to collect and input data. The technology and approach to make it easier for data collection. Investment in iPads or tablet technology available to trainees during training might facilitate data collection. Importance of data collection should be stressed as was done in the pilot. Also, the trainers need to have a binder of all data collection form.
- The SSC program should devise effective methods of translating information shared by parents in their journals as well as other authentic practical solutions they shared in discussions into data for dissemination, validation and analysis.
- Though the men and women were separated, it was recommended that periodically, the men and women groups should interact to provide the gender perspective on some parenting issues both groups struggle with.
- Some of fathers felt some of the barriers to implementing what they learned from the SSC training were societal; for example legal restriction in being active in the child’s life. In these instances, adopting the model of drug courts, where the court might relax the restrictions if a father enrolls in a parenting training course and successfully completes the program, could provide beneficial experiences for the child.
- The confidence gained by the parents during the program needs to be encouraged and sustained with appropriate resources including use of technology to proactively spread the interventions.
- There needs to be annual conference for program alumni to reconnect and an opportunity to share how the program has changed their parenting skills and improved the health of their child or children.

### 3.4. Discussion

The primary aims of this study were to train parents in effective leadership skills that will enable them to: (i) improve parent-child interactions and experiences (ii) increase awareness and understanding of child development, including cognitive, physical, emotional, and social being (iii) improve leadership role in applying quality parenting into daily routines and training their peers on how quality parenting can positively impact the development of children’s behavioral, physical, cognitive and emotional health. We successfully accomplished our primary aims and learned from the parents experience ways to improve the SSC parent leadership curriculum. This resulted in developing two pronged curriculum with a parent leadership curriculum that can be offered separately and as a compliment to the SSC quality parenting curriculum. Emerging findings from our evaluation led to significant improvements being made to the SSC parent leadership curriculum to enhance parents’ effectiveness in facilitating conversations and asking the right questions to transform practical parenting behaviors in the community, as a positive social determinant of health, for broader application.

This analysis suggests that parents coming together every week to create a supportive network improves their own mental health conditions and reduces social isolation. This result indicates that SSC parenting education facilitated by peers in a social supportive environment improves both quality parenting knowledge and skills, in addition to flourishing mental health conditions among participants. 

*SSC Model and Conceptual Framework*: In examining the theoretical basis of the SSC parenting curriculum it utilizes three of the commonly used theories in designing, developing and implementing parenting curricula particularly if psychological significance of the parent-child relationship is focal to effective parenting. Social learning theory and attachment theory have been both cited in contemporary research on parent-child relationships [35]. Since several leading practitioners have expanded the social learning model to incorporate considerations of the parent’s social setting that might be a major contributor to poor parenting; its application to SSC curriculum is appropriate given that the target population is low SES and have several environmental and social factors that might hinder their effective parenting skills. SSC confronts these barriers to effective parenting by providing knowledge and help the parents in a non-judgmental way to devise strategies to provide excellent parenting. SSC also assist the parents through resource mapping and referral to change some of the environmental factors to ensure that knowledge acquired and skills gained are applied. 

Attachment Theory broadens the parent-child relationships by incorporating etiology, cognitive psychology and control systems. The focus of the neurobiology of brain and child development of the SSC curriculum accounts for the cognitive psychology aspect. SSC parenting model stresses the importance of providing safety and protection for the child, thus protecting the child from harm and creating for the child, a sense of emotional security. 

Attribution Theory accounts for the processes by which individuals explain the causes of behavior and events. To effect change, the parents needed to have a safe and stimulating environment that they could talk about the causes of the current parenting practices, the behavior in the context of their relationships with their children and the events that have occurred in their lives to date. This theory also is utilized in SSC under the premise that parents are the child’s first teacher. Hence, parents then see the need to be active in the child’s life and to be the best role model that they can conceivably be.

Masten and Coatsworth [35] catalogue a number of studies that suggest that parent-child relationship quality could lead to the following ills: aggressive behavior and delinquency; depression, anxiety and internalizing problems; cognitive and educational outcomes; social competence and peer relationships—affected mainly by the quality of child-parent attachment in infancy and early childhood; self-esteem and identification; and general health and biological development. SSC was designed to mitigate these consequences of poor parenting with the purpose of mitigating health disparities in children and later in adulthood. By improving child-parenting relationships with a community-based participatory model parenting curriculum, SSC addresses parenting as a public health matter, which can be effectively addressed by research, education and policy. The need to engage the community to design, develop and implement SSC parenting curriculum is critical particularly given the significant impact and role that CBPR is having on the conduct of research. Wallerstein and Duran [36] indicated that CBPR has emerged as a transformative research paradigm that bridges the gap between science and practice through community engagement and social action to increase health equity. CBPR is an integral part of translational research since the voice and intellect of the community is factored in the planning and conduct of the research. 

Though the initial level of the knowledge of brain and child development was low, it was what was expected in the target population. However, since the curriculum uses train-the-trainer model, knowledge reinforcement is more likely to increase knowledge to expected levels. The fact that the parents were able to discuss the information as a conversational piece also aids the process of application of knowledge since we know that knowledge acquisition does not always translate to behavior modification [37]. A fidelity assessment was part of the evaluation process. 

Except for a few sections of the curriculum, there was no gender difference in knowledge. In terms of mental health assessment, we found that the first generation of SSC parents reported a high mental health flourishing state. They seem to be highly motivated and positively strong individuals who are seeking opportunities to improve their parenting skills in raising smart and secure children. To further test the extent to which the peer delivery of SSC in a social supportive group impacts mental health of participants, we added two questions from PHQ-2 standard instrument for measuring depression. This we think will help us to determine if depressive symptoms will reduce as well as mental health condition increase in our current demonstration study groups. Studies have showed that people in the fourth quadrant of Keyes’ [33] mental health continuum have a high level of mental illness but can also exhibit some degree of flourishing (*i.e.*, exhibit a level of resilience and coping factors) [38]. As a result, it is pertinent to assess the extent to which SSC could reduce depressive symptoms of parents participating in social supportive quality parenting learning experience. 

Though great strives were made with parents in terms of being leaders first in their household, and then in their community, we learned that intentional development of the parent mentors and leaders will activate deep behavior change that will last and result in authentic and effective leadership. We observed through the discussions in the classes and during the graduation ceremonies that the parents’ leadership skills were emerging and they reported tremendous growth in knowledge of what they can do to better promote their child’s brain development and social emotional health. To borrow the name of one of the tribes, the Butterfly Effect, we observed over the course of the training that the parents were undergoing a metamorphosis starting from a larva to a butterfly. 

### 3.5. Implications of Study Findings

Findings from this study will contribute a translational culturally relevant parenting education model. A model that prepares parents from a vulnerable state in life to emerging leaders who can transform their parenting behaviors to be more effective parents and improve their children’s cognitive, physical and emotional development and health. These improvements will lead to reduction in existing health disparities of children. This study also suggests an emerging practice-based delivery method “conversepedia” holds promise as viable natural learning experience that can transform behaviors, active authentic leadership and improve health outcomes of children and their parents. Also, the use of social supportive learning approach leads to social inclusion and can promote mental health and wellbeing. In light of the limitations inherent in pilot studies, further studies to demonstrate the efficacy of the use of parent leadership and conversepedia approaches for facilitating behavior changes and lasting improvements that could result in cultural and lifestyle change are necessary and important. 

## 4. Conclusions

“Conversepedia” is an effective method for: increasing parenting skills that modify behavior; and delivering a fully integrated community engagement model intervention that utilizes CBPR. Adults learn more through a multi-sensory curriculum; and are likely to change their behaviors when they co-create learning experiences and deliver them in natural format. Parents do matter and when provided education and options they can choose positive influence in their lives and are capable of reversing negative peer influence. Resnick and colleagues [39] demonstrated through the use of longitudinal research that parents have a greater impact on their teens than previously thought. 
